# A New Family of DNA Viruses Causing Disease in Crustaceans from Diverse Aquatic Biomes

**DOI:** 10.1128/mBio.02938-19

**Published:** 2020-01-14

**Authors:** Kuttichantran Subramaniam, Donald C. Behringer, Jamie Bojko, Natalya Yutin, Abigail S. Clark, Kelly S. Bateman, Ronny van Aerle, David Bass, Rose C. Kerr, Eugene V. Koonin, Grant D. Stentiford, Thomas B. Waltzek

**Affiliations:** a Department of Infectious Diseases and Immunology, College of Veterinary Medicine, University of Florida, Gainesville, Florida, USA; b Emerging Pathogens Institute, Gainesville, Florida, USA; c School of Forest Resources and Conservation, Program in Fisheries and Aquatic Sciences, University of Florida, Gainesville, Florida, USA; d National Center for Biotechnology Information, National Library of Medicine, National Institutes of Health, Bethesda, Maryland, USA; e Elizabeth Moore International Center for Coral Reef Research & Restoration, Mote Marine Laboratory, Summerland Key, Florida, USA; f International Centre of Excellence for Aquatic Animal Health, Centre for Environment Fisheries and Aquaculture Science (Cefas), The Nothe, Dorset, United Kingdom; g Centre for Sustainable Aquaculture Futures, University of Exeter, Exeter, United Kingdom; Columbia University College of Physicians and Surgeons

**Keywords:** Crustacea, genome degradation, large nucleocytoplasmic DNA viruses, low-complexity sequences, virus evolution

## Abstract

Panulirus argus virus 1 (PaV1) is the only known virus infecting the Caribbean spiny lobster (Panulirus argus) from the Caribbean Sea. Recently, related viruses, Dikerogammarus haemobaphes virus 1 (DhV1) and Carcinus maenas virus 1 (CmV1), have been detected in the demon shrimp (Dikerogammarus haemobaphes) and the European shore crab (Carcinus maenas), respectively, from sites in the United Kingdom. The virion morphology of these crustacean viruses is similar to that of iridoviruses. However, unlike iridoviruses and other nucleocytoplasmic large DNA viruses (NCLDVs), these viruses complete their morphogenesis in the host cell nucleus rather than in the cytoplasm. To date, these crustacean viruses have remained unclassified due to a lack of genomic data. Using an Illumina MiSeq sequencer, we sequenced the complete genomes of PaV1, CmV1, and DhV1. Comparative genome analysis shows that these crustacean virus genomes encode the 10 hallmark proteins previously described for the NCLDVs of eukaryotes, strongly suggesting that they are members of this group. With a size range of 70 to 74 kb, these are the smallest NCLDV genomes identified to date. Extensive gene loss, divergence of gene sequences, and the accumulation of low-complexity sequences reflect the extreme degradation of the genomes of these “minimal” NCLDVs rather than any direct relationship with the NCLDV ancestor. Phylogenomic analysis supports the classification of these crustacean viruses as a distinct family, “Mininucleoviridae,” within the pitho-irido-Marseille branch of the NCLDVs.

## Abstract (precis)

Recent genomic and metagenomic studies have led to a dramatic expansion of the known diversity of nucleocytoplasmic large DNA viruses (NCLDVs) of eukaryotes, which include giant viruses of protists and important pathogens of vertebrates, such as poxviruses. However, the characterization of viruses from nonmodel hosts still lags behind. We sequenced the complete genomes of three viruses infecting crustaceans, the Caribbean spiny lobster, demon shrimp, and European shore crab. These viruses have the smallest genomes among the known NCLDVs, with losses of many core genes, some of which are shared with iridoviruses. The deterioration of the transcription apparatus is compatible with microscopic and ultrastructural observations indicating that these viruses replicate in the nucleus of infected cells rather than in the cytoplasm. Phylogenomic analysis indicates that these viruses are sufficiently distinct from all other NCLDVs to justify the creation of a separate family, for which we propose the name “Mininucleoviridae” (i.e., small viruses reproducing in the cell nucleus).

## INTRODUCTION

The nucleocytoplasmic large DNA viruses (NCLDVs) are a diverse assemblage of viruses infecting a range of unicellular eukaryotes and animals (1). The NCLDVs share an ancient origin and encompass seven formally recognized families, including the *Poxviridae*, *Asfarviridae*, *Iridoviridae*, *Ascoviridae*, *Phycodnaviridae*, *Mimiviridae*, and *Marseilleviridae*, along with the proposed family “Pithoviridae” and some unclassified groups, such as pandoraviruses and medusaviruses (1). They replicate exclusively within the cytoplasm (e.g., *Poxviridae*) or involve both the nucleus and the cytoplasm (e.g., *Iridoviridae*) of the host cell (2). Based on their large virions and genomes, a suite of conserved genes, and a unique replication scheme, the order “Megavirales” has been proposed to unite the NCLDV families (3).

Several potential NCLDVs have been identified from crustacean hosts, but these do not appear to replicate in the cytoplasm (as generally expected of NCLDVs) and remain to be studied systematically (4–7). These viruses develop within the nucleus of crustacean host hemocytes and hemopoietic tissues, resulting in anemia and subsequent death. Here, we provide genomic data for three crustacean-infecting viral pathogens that infect hosts from three different aquatic niches: the Caribbean spiny lobster, Panulirus argus (Palinuridae), from a tropical marine environment; the demon shrimp, Dikerogammarus haemobaphes (Amphipoda), from a riverine freshwater environment; and the European shore crab, Carcinus maenas (Brachyura), from a coastal marine/brackish environment. These genomic data are coupled with preexisting information on the microscopic and ultrastructural pathology induced by these viruses (5–7). Using combined genomic, microscopic, and ultrastructural data, we propose the creation of a new virus family, the “Mininucleoviridae,” within the NCLDV group.

The Caribbean spiny lobster is a shallow (<100 m) water marine species ranging from the coast of North Carolina (United States) through to northern South America, supporting important commercial fisheries across its range. All life stages (excluding the larval stage) are susceptible to “Panulirus argus virus 1” (PaV1). PaV1 was discovered in P. argus from the Florida Keys in 2000 (5) and remains the only naturally occurring virus reported from any lobster species. Infected lobsters become increasingly lethargic as they develop acute signs of infection, which often correlates with their hemolymph turning from clear to milky white (8). PaV1 primarily infects circulating hemocytes (hyalinocytes and semigranulocytes) and hemopoietic tissues, ultimately causing mortality due to anemia and metabolic exhaustion (5). The prevalence of infection is inversely related to lobster size, with the smallest juvenile lobsters (<20-mm carapace length) suffering the highest infection rates (5, 9).

Infections with PaV1 have been reported from most of the range of *P. argus*, including Florida, the Gulf of Mexico, and throughout the Caribbean (5, 10–12). The prevalence of clinical infections in the Florida Keys (United States) and the Yucatan Peninsula (Mexico) can reach 70% at some locations, not including subclinical PCR-based detection estimates, which can be an order of magnitude higher than clinical estimates (13; D. C. Behringer, unpublished data). Clinical infections are 100% fatal, so considering prevalence estimates from the Florida Keys, this corresponds to an estimated 24% juvenile lobster mortality due to PaV1 infection prior to reaching maturity (12). Apparently healthy adult lobsters often test positive for PaV1 using molecular assays (PCR assays) but do not develop gross or microscopic lesions as observed in clinically affected animals (14, 15). PaV1 also has remarkable effects on lobster ecology, stemming from the ability of healthy lobsters to detect and avoid lobsters infected with PaV1 (16). This avoidance behavior is driven by chemosensory cues found in the urine of infected individuals (17) and appears to be an efficient mechanism of “behavioral immunity” (18, 19).

The second virus in our study, previously termed “Dikerogammarus haemobaphes bi-facies-like virus” (DhbflV) but referred to here as “Dikerogammarus haemobaphes virus 1” (DhV1), has been observed during laboratory experimentation with the amphipod D. haemobaphes (6). This species originates from the Ponto-Caspian region and is now present in the United Kingdom after following an invasion route through most of central Europe (6). This virus has been associated with host mortality (∼30% mortality of laboratory animals) and appears to exhibit a population-regulating effect in the nonnative population range, potentially reducing the impact of the host therein (6). The third virus, previously known as herpes-like virus (HLV) but referred to here as “Carcinus maenas virus 1” (CmV1), has morphological similarity to PaV1 and DhV1; is present at a 3.7% prevalence (during summer months) in populations of the European shore crab, *Carcinus maenas*, in the United Kingdom (7, 20); and causes a pathology similar to that observed in PaV1-infected spiny lobsters (7). This crab species is globally invasive; however, host populations outside Europe do not appear to harbor the virus (7). In the present study, we sequenced the genomes of PaV1, DhV1, and CmV1, and compared them to the genomes of viruses currently known to be associated with the NCLDV group.

## RESULTS AND DISCUSSION

**FIG 1:**
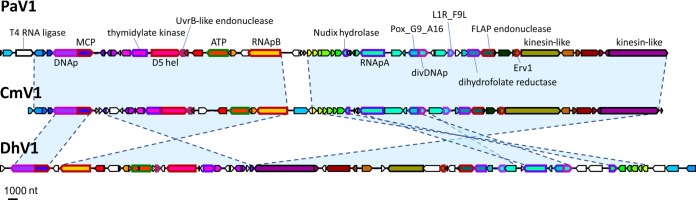
Genome maps of the crustacean viruses PaV1 (Panulirus argus virus 1), DhV1 (Dikerogammarus haemobaphes virus 1), and CmV1 (Carcinus maenas virus 1). Predicted orthologous genes are marked with the same colors. nt, nucleotides. Gene abbreviations: DNAp, family B DNA polymerase; MCP, major capsid protein; D5 hel, D5-like primase-helicase; ATP, packaging ATPase; RNApB, DNA-dependent RNA polymerase subunit RPB2; RNApA, DNA-dependent RNA polymerase subunit RPB1; Pox_G9-A16, poxvirus entry-fusion complex G9-A16; divDNAp, divergent family B DNA polymerase; L1R_F9L, lipid membrane protein of large eukaryotic DNA viruses; Erv1, sulfhydryl/thiol oxidoreductase (ERV1/ALR/poxvirus E10 family).

**FIG 2:**
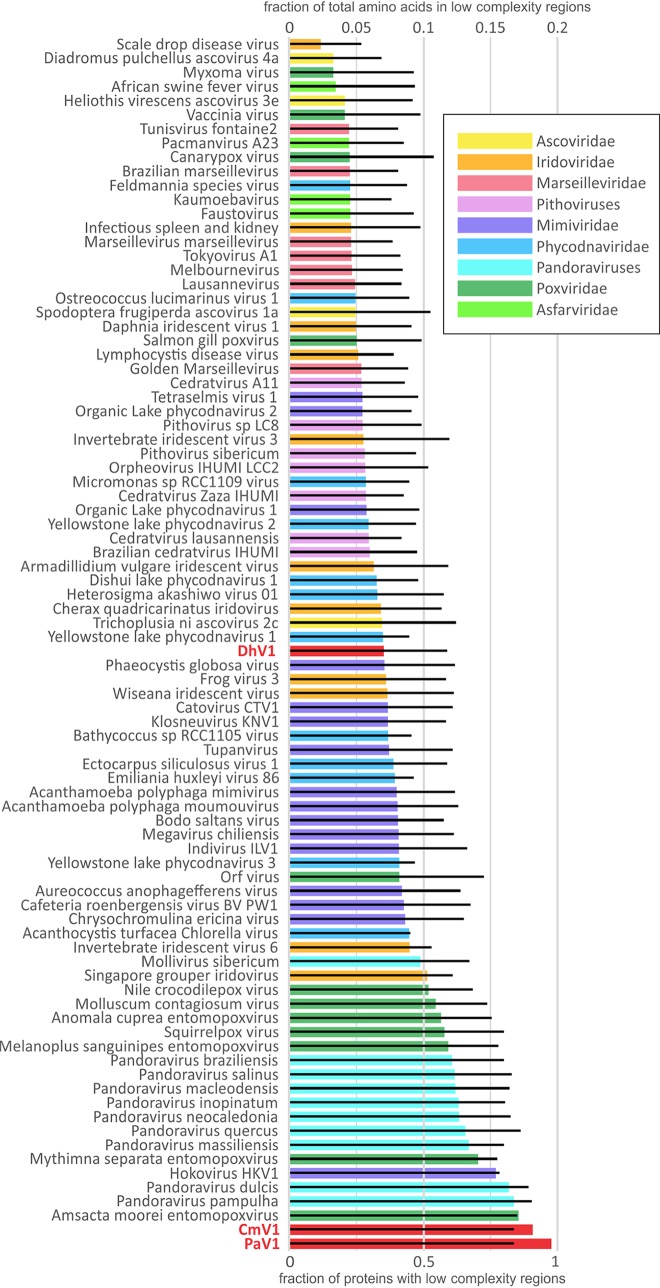
Relative abundances of low-complexity sequences as a fraction of the total amino acid content contained in low-complexity regions (colored bars) and as a fraction of proteins with detectable low-complexity regions (black bars). Virus abbreviations: PaV1, Panulirus argus virus 1; DhV1, Dikerogammarus haemobaphes virus 1; CmV1, Carcinus maenas virus 1.

PaV1, DhV1, and CmV1 have genomes of 70,886 bp, 73,581 bp, and 66,929 bp, with 61, 61, and 62 predicted protein-coding genes, respectively. The predicted proteins of the three viruses were grouped into 48 clusters of apparent orthologs (see Materials and Methods for the details). The genomes of PaV1, DhV1, and CmV1 were shown to encode 13, 20, and 13 unique proteins, respectively. Extensive protein sequence similarity searches using PSI-BLAST against the National Center for Biotechnology Information (NCBI) nonredundant database or searches against the clusters of orthologous NCLDV genes (NCVOGs) (21) as well as sensitive profile-profile searches using HHPred, combined with predictions of structural features, yielded relevant information for 26 clusters of orthologs and an additional PaV1 protein (see Table S1 in the supplemental material) (complete HHpred results are available at ftp://ftp.ncbi.nih.gov/pub/yutinn/Crustacean_viruses_2018/CLSV_HHpred_raw_output/). Taken together, these comparisons of the gene repertoires indicate a high level of gene content conservation among these three crustacean-infecting viruses. Furthermore, a comparison of the annotated genome maps shows a near-complete conservation of synteny between PaV1 and CmV1, whereas the more distant DhV1 shares two large syntenic segments with the other two viruses albeit in inverted orientations (Fig. 1). A distinctive feature of the proteins encoded by these viruses (primarily PaV1 and CmV1) is the high content of low-complexity (repetitive and quasirepetitive) regions (LCRs) compared to other NCLDVs (Fig. 2). Among the 40 clusters of orthologous proteins in which all 3 crustacean viruses are represented, 18 contained LCRs in all three proteins, 15 included two proteins with LCRs, and 7 included one LCR-containing protein (Table S1), attesting to the high prevalence of LCRs in these viruses. The LCRs are typically located either in terminal regions of the respective proteins or between conserved domains, as illustrated in Fig. S1 for three examples of orthologous protein clusters. The accumulation of LCRs is likely to reflect weak selection in small populations in the course of reductive evolution (22, 23). A similar phenomenon is observed in many parasitic bacteria with small, degraded genomes (24). The LCRs in virion proteins could substantially contribute to antigenic variation (25).

10.1128/mBio.02938-19.1 FIG S1 Low-complexity segments in proteins of the 3 members of the proposed family “Mininucleoviridae.” The content of low-complexity sequences is illustrated by the alignment of 3 proteins that are conserved in most of the NCLDVs. The low-complexity segments in the proteins of mininucleoviruses are highlighted in each alignment. Download FIG S1, PDF file, 0.6 MB. Copyright © 2020 Subramaniam et al. 2020 Subramaniam et al. This content is distributed under the terms of the Creative Commons Attribution 4.0 International license.

10.1128/mBio.02938-19.2 TABLE S1 Complete annotation of the genes of the 3 members of the proposed family “Mininucleoviridae.” Download Table S1, XLSX file, 5.0 MB. Copyright © 2020 Subramaniam et al. 2020 Subramaniam et al. This content is distributed under the terms of the Creative Commons Attribution 4.0 International license.

Of those genes that are conserved among the three crustacean viruses, 13 belong to NCVOGs; i.e., they have identifiable orthologs in other NCLDVs (Table S1). Among the 20 core genes with the highest representation among the NCLDVs, these viruses retain 10 (Table 1). They encode the major capsid protein (MCP) and the packaging ATPase; the key proteins involved in virus replication, namely, DNA polymerase (DNAP), primase-helicase, and FLAP endonuclease; three RNA polymerase (RNAP) subunits; and two disulfide-bonded structural proteins along with the disulfide-thiol oxidoreductase that is required for their biogenesis (Table 1). The conservation of the core NCLDV genes clearly indicates that these crustacean viruses belong to the NCLDV group. However, they have the smallest genomes among all known NCLDVs and appear to have undergone substantial reductive evolution. In particular, the machinery for transcription and mRNA modification is drastically reduced. Missing are the rest of the RNAP subunits, all transcription factors, and the capping enzyme, and unlike the great majority of the NCLDVs, the crustacean viruses encode no helicases other than the primase-helicase, which is essential for replication, or topoisomerases (Table 1). These viruses also lack enzymes for nucleotide metabolism except for a putative thymidylate kinase. Some of these conspicuous gaps among the NCLDV core genes in these viruses are shared with subsets of *Iridoviridae* representatives, the NCLDV family previously thought to possess the smallest genomes.

**TABLE 1:** Core genes of nucleocytoplasmic large DNA viruses present and absent from mininucleoviruses^*a*^

Virus	No. of genes																				
	MCP (NCVOG0022)	ATP (NCVOG0249)	Erv1 (NCVOG0052)	DNAP (NCVOG0038)	D5_hel (NCVOG0023)	TopoII (NCVOG0037)	FLAP (NCVOG1060)	A18_hel (NCVOG0076)	VLTF3 (NCVOG0262)	VLTF2 (NCVOG1164)	VETF (NCVOG0261)	TFIIB (NCVOG1127)	TFIIB (NCVOG0272)	RNAPA (NCVOG0274)	RNAPB (NCVOG0271)	RNAP5 (NCVOG0273)	capG (NCVOG1117)	capM (NCVOG1117)	NUDIX (NCVOG0236)	RRL (NCVOG1353)	RRs (NCVOG0276)
**PaV1**	1	1	1	2	1	0	1	0	0	0	0	0	0	1	1	1	0	0	1	0	0
**DhV1**	1	1	1	2	1	0	1	0	0	0	0	0	0	1	1	1	0	0	1	0	0
**CmV1**	1	1	1	2	1	0	1	0	0	0	0	0	0	1	1	1	0	0	1	0	0

Asco- and iridoviruses																					
Heliothis_virescens_ascovirus_3e	1	1	1	1	1	0	1	0	1	1	1	0	0	1	1	1	0	0	0	0	0
Spodoptera_frugiperda_ascovirus_1a	1	1	1	1	1	0	1	0	1	1	1	0	0	1	1	1	0	0	0	0	0
Trichoplusia_ni_ascovirus_2c	2	1	1	1	1	0	1	0	1	1	1	0	1	1	1	1	0	0	0	0	1
Diadromus_pulchellus_ascovirus_4a	1	1	1	1	2	0	0	0	1	1	1	0	1	1	1	1	0	0	1	1	1
Armadillidium_vulgare_iridescent_virus	1	1	1	1	1	1	1	1	1	1	1	0	1	1	1	1	0	0	1	1	1
Invertebrate_iridescent_virus_6	1	1	1	1	1	1	1	1	1	1	1	0	1	1	1	1	0	0	1	1	1
Daphnia_iridescent_virus_1	1	1	1	1	1	0	1	1	1	1	1	0	1	1	1	0	1	0	0	1	1
Wiseana_iridescent_virus	1	1	1	1	1	1	1	1	1	1	1	0	1	1	1	1	0	0	2	1	1
Invertebrate_iridescent_virus_3	1	1	1	1	1	1	1	1	1	1	1	0	1	1	1	1	0	0	2	1	1
Cherax_quadricarinatus_iridovirus	1	1	1	1	1	1	1	1	1	1	1	0	1	1	1	1	0	0	1	0	0
Infectious_spleen_and_kidney	1	1	1	1	1	0	1	0	1	0	1	0	1	1	1	0	1	0	0	0	1
Scale_drop_disease_virus	1	1	1	1	1	0	1	0	1	0	1	0	1	1	1	0	1	0	0	0	1
Lymphocystis_disease_virus	1	1	1	1	1	0	1	0	1	1	1	0	1	1	1	1	0	0	1	1	1
Frog_virus_3	1	1	1	1	1	0	1	1	1	1	1	0	1	1	1	1	0	0	0	1	1
Singapore_grouper_iridovirus	1	1	1	1	1	0	1	1	1	1	1	0	1	1	1	1	0	0	0	1	1

Pithoviruses																					
Cedratvirus_Zaza_IHUMI	1	0	1	1	1	1	1	1	1	1	1	1	1	1	1	1	1	1	0	1	1
Cedratvirus_lausannensis	1	0	1	1	1	1	1	1	1	1	1	1	1	1	1	1	1	1	0	1	1
Cedratvirus_A11	1	0	1	1	1	1	1	1	1	1	1	1	1	1	1	1	1	1	0	1	1
Brazilian_cedratvirus_IHUMI	1	0	1	1	1	1	1	1	1	1	1	1	1	1	1	1	1	1	0	1	1
Pithovirus_sibericum	1	0	1	1	1	1	1	1	1	1	1	1	1	1	1	1	1	1	1	1	1
Orpheovirus_IHUMI_LCC2	1	0	1	1	1	1	1	1	1	0	1	1	1	1	1	1	1	0	2	1	1

Marseilleviruses																					
Golden_Marseillevirus	1	1	1	1	1	1	1	1	1	1	0	1	0	1	1	1	0	1	1	1	1
Brazilian_marseillevirus	1	1	1	1	1	1	1	1	1	1	1	1	1	1	1	1	1	1	2	1	1
Lausannevirus	1	1	1	1	1	1	1	1	1	1	1	1	1	1	1	1	1	1	2	1	1
Tunisvirus_fontaine2	1	1	1	1	1	1	1	1	1	1	1	1	1	1	1	1	1	1	2	1	1
Tokyovirus_A1	1	1	1	1	1	1	1	1	1	1	1	1	1	1	1	1	1	1	2	1	1
Marseillevirus_marseillevirus	1	1	1	1	1	1	1	1	1	1	1	1	1	1	1	1	1	1	2	1	1
Melbournevirus	1	1	1	1	1	1	1	1	1	1	1	1	1	1	1	1	1	1	1	1	1
Mimiviruses																					
Tupanvirus	5	4	3	1	2	1	1	1	1	1	1	2	1	1	1	1	1	1	2	1	1
Acanthamoeba_polyphaga_mimivirus	4	2	2	1	1	1	1	1	1	1	1	1	1	1	1	1	1	1	1	1	1
Acanthamoeba_polyphaga_moumouvirus	4	2	2	1	1	1	1	1	1	1	1	1	1	1	1	1	1	1	2	1	1
Megavirus_chiliensis	4	2	2	1	1	1	1	1	1	1	1	1	1	1	1	1	2	1	2	1	1
Cafeteria_roenbergensis_virus_BV_PW1	4	1	2	1	1	1	1	1	1	1	1	1	1	1	1	1	1	1	1	1	1
Hokovirus_HKV1	6	2	3	1	3	1	1	1	1	1	1	1	1	1	1	1	1	2	1	1	1
Bodo_saltans_virus	4	1	2	1	18	1	1	1	1	1	1	1	1	1	1	1	1	1	2	1	1
Catovirus_CTV1	5	2	2	1	2	2	1	1	1	1	1	1	1	1	1	1	3	2	1	1	1
Indivirus_ILV1	6	2	3	1	2	1	1	1	1	1	1	1	1	1	1	1	2	1	1	1	1
Klosneuvirus_KNV1	8	3	4	1	2	1	1	1	1	1	1	1	1	1	1	1	1	1	3	1	1
Tetraselmis_virus_1	1	1	1	1	2	1	0	1	1	1	0	1	1	1	1	1	1	1	1	1	1
Aureococcus_anophagefferens_virus	2	1	1	1	2	1	0	1	1	1	0	1	1	1	1	2	1	1	0	1	1
Organic_Lake_phycodnavirus_1	2	1	3	1	1	1	0	1	1	1	0	1	1	1	1	1	1	1	2	1	1
Organic_Lake_phycodnavirus_2	2	1	1	1	1	1	0	1	1	1	1	1	1	1	1	1	1	1	1	1	1
Chrysochromulina_ericina_virus	4	1	3	1	1	1	1	1	1	1	1	1	1	1	1	1	1	1	1	1	1
Phaeocystis_globosa_virus	2	2	3	1	1	1	0	1	1	1	1	1	1	1	1	1	1	1	1	1	1

Phycodna- and pandoraviruses																					
Heterosigma_akashiwo_virus_01	1	1	0	1	1	1	1	0	1	1	0	1	1	0	0	0	1	1	0	0	0
Acanthocystis_turfacea_Chlorella_virus	5	1	1	1	1	1	0	1	1	1	0	1	1	0	0	0	1	0	0	1	1
Yellowstone_lake_phycodnavirus_1	5	1	2	1	1	1	0	1	1	1	0	1	1	0	0	0	1	1	1	1	1
Yellowstone_lake_phycodnavirus_2	6	1	2	1	1	2	0	0	1	0	0	1	0	0	0	0	1	1	1	1	1
Yellowstone_lake_phycodnavirus_3	7	1	0	0	1	0	0	1	2	1	0	1	1	0	0	0	1	1	1	2	1
Dishui_lake_phycodnavirus_1	7	1	0	1	1	1	0	1	1	1	0	1	1	0	0	0	1	1	1	1	1
Bathycoccus_sp_RCC1105_virus	7	1	0	1	1	1	0	1	1	1	0	1	1	0	0	0	1	1	1	1	1
Ostreococcus_lucimarinus_virus_1	8	1	0	1	1	1	0	1	1	1	0	1	1	0	0	0	1	1	1	1	1
Micromonas_sp_RCC1109_virus	8	1	0	1	1	1	0	1	1	1	0	1	1	0	0	0	1	1	1	1	1
Ectocarpus_siliculosus_virus_1	1	1	1	1	1	0	0	1	1	1	0	0	0	0	0	0	0	0	0	1	1
Feldmannia_species_virus	1	1	1	1	1	0	0	0	1	1	0	0	0	0	0	0	0	0	0	1	1
Emiliania_huxleyi_virus_86	1	1	1	1	1	1	1	1	1	1	0	0	1	1	1	1	1	0	0	1	1
Mollivirus_sibericum	1	1	2	1	1	0	1	1	1	1	0	0	1	1	1	1	1	0	0	0	0
Pandoravirus_macleodensis	0	3	2	1	1	0	0	1	1	1	0	0	1	1	1	1	1	1	0	1	1
Pandoravirus_neocaledonia	0	3	2	1	1	0	0	1	1	1	0	0	1	1	1	1	1	1	0	1	1
Pandoravirus_salinus	0	5	2	1	1	0	0	1	1	1	0	0	1	1	1	1	1	1	1	1	1
Pandoravirus_dulcis	0	2	2	1	1	0	0	1	1	1	0	0	1	1	1	1	1	1	0	1	1
Pandoravirus_quercus	0	2	2	1	1	0	0	1	1	1	0	0	1	1	1	1	1	1	0	1	1
Pandoravirus_inopinatum	0	2	2	1	1	0	0	1	1	1	0	0	1	1	1	1	1	1	0	1	1
Asfarviruses																					
Pacmanvirus_A23	1	1	0	1	1	1	1	1	1	1	1	1	1	1	1	1	1	1	2	1	1
Faustovirus	1	1	0	1	1	1	1	1	1	1	1	1	1	1	1	1	1	1	1	1	1
African_swine_fever_virus	1	1	1	1	1	1	0	1	1	1	1	1	1	1	1	1	1	1	1	1	1
Kaumoebavirus	1	1	1	1	1	1	1	1	1	1	1	1	1	1	1	1	1	1	1	1	1

Poxviruses																					
Amsacta_moorei_entomopoxvirus	1	1	1	1	1	0	1	1	1	1	1	0	0	1	1	0	1	1	0	0	0
Mythimna_separata_entomopoxvirus	1	1	1	1	1	0	1	1	1	1	1	0	0	1	1	0	1	1	1	0	0
Anomala_cuprea_entomopoxvirus	1	1	1	1	1	0	1	1	1	1	1	0	0	1	1	0	1	1	1	0	0
Melanoplus_sanguinipes_entomopoxvirus	1	1	1	1	1	0	1	1	1	1	1	0	0	1	1	0	1	1	1	0	0
Salmon_gill_poxvirus	1	1	1	1	1	0	1	1	1	1	1	0	1	1	1	0	1	1	1	0	0
Nile_crocodilepox_virus	1	1	1	1	1	1	1	1	1	1	1	0	1	1	1	0	1	1	2	0	0
Canarypox_virus	1	1	1	1	1	0	1	1	1	1	1	0	1	1	1	0	1	1	2	0	1
Molluscum_contagiosum_virus	1	1	1	1	1	0	1	1	1	1	1	0	1	1	1	0	1	1	2	0	0
Myxoma_virus	1	1	1	1	1	0	1	1	1	1	1	0	1	1	1	0	1	1	2	0	1
Vaccinia_virus	1	1	1	1	1	0	1	1	1	1	1	0	1	1	1	0	1	1	2	1	1
Squirrelpox_virus	1	1	1	1	1	0	1	1	1	1	1	0	1	1	1	0	1	1	2	0	0
Orf_virus	1	1	1	1	1	0	1	1	1	1	1	0	1	1	1	0	1	1	1	0	0

a Virus abbreviations: PaV1, Panulirus argus virus 1; DhV1, Dikerogammarus haemobaphes virus 1; CmV1, Carcinus maenas virus 1. Core NCLDV protein abbreviations: MCP, NCLDV major capsid protein (NCVOG0022); ATP, A32-like packaging ATPase (NCVOG0249); Erv1, Erv1/Alr family disulfide (thiol) oxidoreductase (NCVOG0052); DNAP, family B DNA polymerase (NCVOG0038); D5_hel, D5-like helicase-primase (NCVOG0023); TopoII, DNA topoisomerase II (NCVOG0037); FLAP, FLAP-like endonuclease XPG (NCVOG1060); A18_hel, A18-like helicase (NCVOG0076); VLTF3, poxvirus late transcription factor VLTF3 (NCVOG0262); VLTF2, poxvirus late transcription factor VLTF2 (NCVOG1164); VETF, poxvirus early transcription factor VETF (NCVOG0261); TFIIB, transcription initiation factor IIB (NCVOG1127); TFIIS, transcription factor S-II (TFIIS) (NCVOG0272); RNAPA, DNA-directed RNA polymerase subunit alpha (NCVOG0274); RNAPB, DNA-directed RNA polymerase subunit beta (NCVOG0271); RNAP5, DNA-directed RNA polymerase subunit 5 (NCVOG0273); capG, mRNA capping enzyme, guanylyltransferase (NCVOG1117); capM, mRNA capping enzyme, methyltransferase (NCVOG1117); NUDIX, Nudix hydrolase (NCVOG0236); RRL, ribonucleoside diphosphate reductase, large subunit (NCVOG1353); RRs, ribonucleoside diphosphate reductase, beta subunit (NCVOG0276).

**FIG 3:**
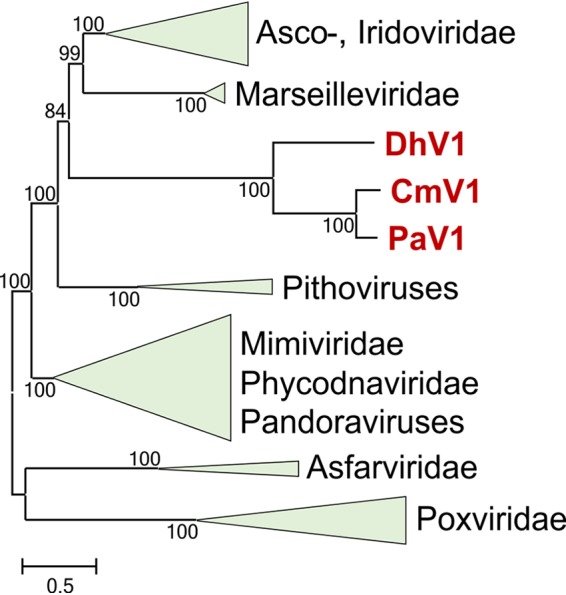
Phylogenetic tree of nucleocytoplasmic large DNA viruses (NCLDVs). The maximum likelihood (ML) tree depicting the relationship of the viruses PaV1, DhV1, and CmV1 to the rest of the NCLDVs is based on the concatenated amino acid sequence alignments of three core genes (major capsid protein, DNA polymerase, and primase-helicase). The bootstrap values are indicated at each node, and the branch lengths reflect the number of inferred substitutions, as indicated by the bar. Virus abbreviations: PaV1, Panulirus argus virus 1; DhV1, Dikerogammarus haemobaphes virus 1; CmV1, Carcinus maenas virus 1.

Even among the core genes that are shared with other NCLDVs, there are signs of anomalous acceleration of evolution in these viruses. The MCP, primase-helicase, RNAP alpha subunit, and packaging ATPase all show unusually low sequence similarity to orthologs from other NCLDVs. In most database searches, the hallmark proteins of the crustacean viruses show higher sequence similarity to their orthologs in pitho-, irido-asco-, and marseilleviruses (PIM group) than to those in other NCLDVs. Furthermore, one gene of these crustacean viruses that encodes a predicted restriction endonuclease fold enzyme is shared specifically with the PIM group (Table S1). In a phylogenetic tree constructed from concatenated alignments of three hallmark genes (DNAP, primase-helicase, and MCP), these viruses formed a distinct clade within the PIM group, with convincing bootstrap support (Fig. 3).

Apart from the core NCLDV genes, functions could be predicted for only very few genes of these crustacean viruses. A notable feature is the presence, in all three viruses, of two genes encoding kinesin-like ATPases, one of which contains an apparently inactivated ATPase domain. The kinesin-like proteins might play key roles in the interaction between the viruses and their host cell cytoskeleton (26). Of further note are the highly diverged paralogs of family B DNAP that are encoded by all three viruses, in addition to the main DNAP that is orthologous to those of the other NCLDVs. A gene encoding a Nudix family hydrolase appears to be a bacterial acquisition (all the homologs with significant sequence similarity detectable in database searches are bacterial proteins [Table S1]) and is not directly related to the Nudix hydrolases of other NCLDVs, such as the D9 and D10 proteins of poxviruses, which function as decapping enzymes (27, 28). A distinct RNA ligase, for which a distant viral homolog was detected only in Cafeteria roenbergensis virus (CroV), is encoded by PaV1 alone. Given the high sequence similarity between this PaV1 protein and its animal homologs, it appears most likely that PaV1 and CroV have independently acquired genes for homologous RNA ligases.

The results of the comparative genomic and phylogenetic analyses identify these three crustacean viruses as a previously unknown group of “minimal” NCLDVs. This group does not fall into any of the known NCLDV families and appears to be sufficiently distinct from all other NCLDVs to justify the creation of a separate family, for which we propose the name “Mininucleoviridae.” Nevertheless, phylogenetic analysis (Fig. 3) strongly suggests an affinity of this putative family with the PIM group of the NCLDVs, possibly the irido-asco-Marseille branch. The genome size and the gene content of these viruses resemble those of iridoviruses, with some shared losses of core NCLDV genes. The apparent rapid evolution of these viruses that is manifested in extensive gene loss, divergence of the sequences of the remaining genes, and the accumulation of low-complexity sequences suggests that this virus group evolved in the regime of weak selection, which resulted in extensive genome erosion (22, 23). Therefore, it is difficult to rule out that the deep placement of “Mininucleoviridae” in phylogenetic trees is a long-branch artifact, whereas, in actuality, these viruses could be highly derived descendants of iridoviruses.

**FIG 4:**
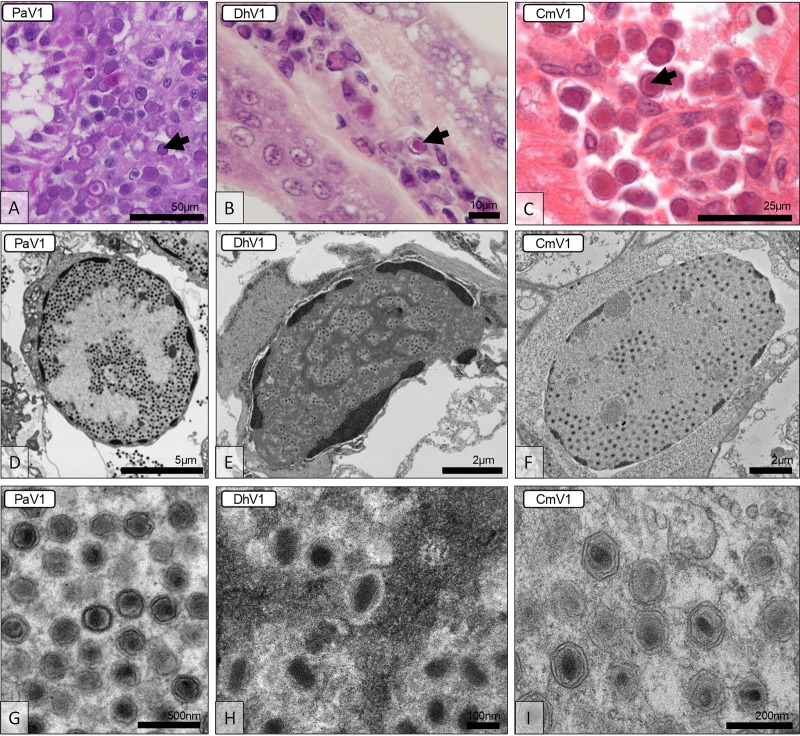
Histological and ultrastructural micrographs of the viruses in *Panulirus argus* (PaV1), *Dikerogammarus haemobaphes* (DhV1), and *Carcinus maenas* (CmV1). (A to C) Histological preparations reveal hemocytes displaying karyomegaly and either basophilic or eosinophilic nuclear inclusions (arrows). (D to F) Transmission electron micrographs reveal enlarged cell nuclei with developing viroplasms and margination of the host chromatin. (G to I) The virion morphologies of the three viruses include an electron-dense genomic core surrounded by a (typically hexagonal) nucleocapsid.

One of the notable aspects of genome degradation in the members of the “Mininucleoviridae” is the loss of most genes for proteins involved in transcription and mRNA maturation. The remaining genes for three RNAP subunits are highly divergent. This deterioration of the transcription apparatus is compatible with microscopic and ultrastructural observations indicating that these viruses replicate in the nucleus of the infected cell (Fig. 4). Histopathological preparations identified the hemopoietic tissues and hemocytes of each host (lobster, shrimp, and crab) as the main seats of infection. Infected cells contained an amorphous viroplasm, apparently restricted to the nucleus (Fig. 4A to C). The infected nucleus exhibited marginated chromatin due to the developing viroplasm (Fig. 4D to F), which was either basophilic (PaV1) or eosinophilic (DhV1 and CmV1) (Fig. 4A to C). By using electron microscopy, the viroplasm was shown to contain masses of virions at various developmental stages (Fig. 4D to F). Virion morphologies of these three crustacean viruses displayed similarities to one another, each possessing hexagonal nucleocapsids of similar diameters (5–7). DhV1 was slightly elliptical, whereas the other two viruses were uniform in width and length (5–7). The genomic core was often observed as being spherical or less structured in PaV1 (Fig. 4G) but was more rod shaped in DhV1 (Fig. 4H) and CmV1 (Fig. 4I). Thus, it seems likely that the transcription machinery of mininucleoviruses has been evolving along the path to elimination as the common ancestor of mininucleoviruses transitioned from cytoplasmic to nuclear replication. This course of evolution parallels the degradation of the transcription apparatus in phycodnaviruses that undergo the early reproduction stages in the nuclei of infected cells and are transported to the cytoplasm at a later stage of infection (29).

**FIG 5:**
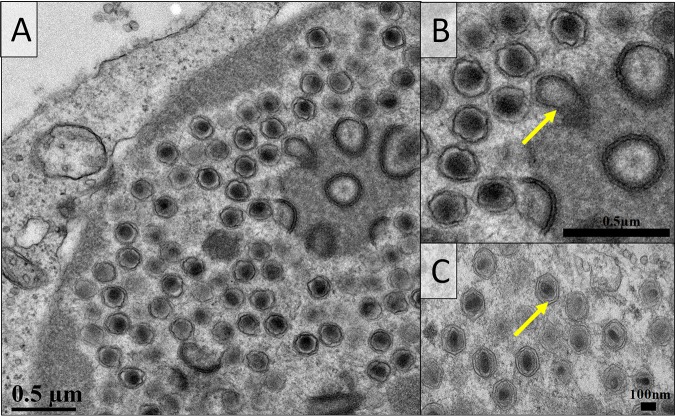
Transmission electron micrographs of Panulirus argus virus 1 (PaV1) (A and B) and Carcinus maenas virus 1 (CmV1) (C). (A) PaV1-infected hemocyte with the viroplasm observed at the periphery of the enlarged nucleus. The assembling virus particles are present with an electron-dense inner membrane surrounded by the nucleocapsid. (B and C) Higher-magnification images permit the visualization of the inner membrane (yellow arrows).

The retention of the disulfide-thiol oxidoreductase is of special interest, emphasizing the importance of this activity in NCLDVs despite dramatic differences in the set of encoded disulfide-bonded proteins. In poxviruses, this protein (E10) is responsible for disulfide bond formation in many proteins of the fusion-entry complex and other structural components of the virion. Mininucleoviruses encode only two proteins that are predicted to form disulfide bonds, namely, a homolog of the G9-A16 subunits of the poxvirus fusion-entry complex and a homolog of the poxvirus myristoylated proteins L1-F9. The presence of these and several uncharacterized membrane proteins (Table S1) implies that mininucleoviruses possess a virion membrane. Indeed, the virions of all three mininucleoviruses included inner membranes resembling the inner membranes present in other NCLDVs (30) (Fig. 5). Mininucleoviruses lacked an external membrane at either the intracellular or the extracellular stage.

**FIG 6:**
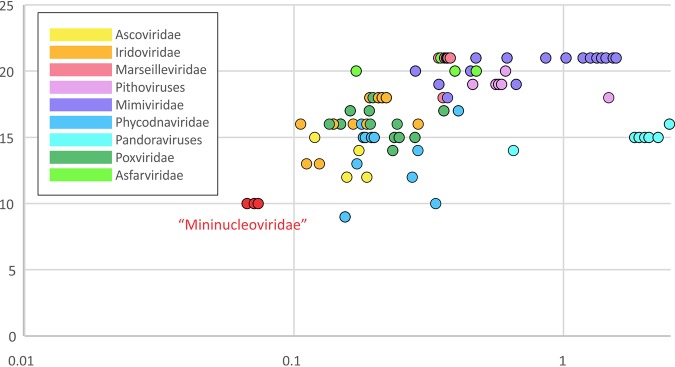
Comparison of the retention and loss of core genes to the genome size of the nucleocytoplasmic large DNA viruses (NCLDVs). The vertical axis shows the number of core genes identified in the genomes of the viruses in each (putative) family of the NCLDVs, and the horizontal axis shows the genome size on a logarithmic scale.

In summary, the three crustacean viruses in the putative family “Mininucleoviridae” represent the minimal form of the NCLDVs discovered so far, with the smallest number of genes. Phylogenomic analysis indicates that this minimalism reflects extreme degradation rather than any direct relationship with the common ancestor of the NCLDVs. Thus, in the overall scenario of NCLDV evolution, this putative new family occupies the opposite end of the spectrum of genome expansion-reduction from giant viruses, such as pandoraviruses and mimiviruses (31). The evolution of the giant viruses appears to have involved extensive gene gain via horizontal gene transfer and duplication, resulting in genome growth (32–34), whereas the evolution of the “Mininucleoviridae” branch was heavily dominated by gene loss. Notably, unlike other viruses that lost some ancestral NCLDV genes but acquired many more new genes (most dramatically, pandoraviruses), in mininucleoviruses, the erosion of the ancestral gene set occurred in parallel with the overall genome shrinking (Fig. 6). The serendipitous discovery of this family of viruses as pathogens of taxonomically diverse crustacean hosts that inhabit freshwater and marine biomes across tropical and temperate climates, without a broadscale screening program targeted at finding them, suggests that there are likely to be many others yet to be discovered. If true, this family may have a potentially significant role as a major mortality driver (and population regulator) for aquatic crustaceans across the globe.

## MATERIALS AND METHODS

### Sample material.

Three juvenile *Panulirus argus* lobsters with clinical PaV1 infections were collected using a hand net from the nearshore hard-bottom habitat of the middle Florida Keys (United States) during June 2015. The lobsters were transported to the Aquatic Pathobiology Laboratory at the University of Florida, where hemolymph samples were drawn. *Dikerogammarus haemobaphes* shrimp were collected from the Carlton Brook freshwater river (Leicestershire, UK) in July 2015. Animals were held in the laboratory for 2 days before dissection and fixation in 99% ethanol. Finally, *Carcinus maenas* crabs were collected from the shoreline at Newtons Cove (Weymouth, Dorset, UK) in August 2015. Animals were anesthetized on ice before dissection, and tissues were fixed in Davidson’s seawater fixative for histology, 2.5% glutaraldehyde in sodium cacodylate buffer for electron microscopy, and 99% ethanol for molecular analysis. For the latter two crustaceans, no gross pathology was observed before dissection, and viral pathology was detected during histological screening.

### DNA extraction and complete genome sequencing.

Total DNA from *P. argu*s hemolymph was extracted using a DNeasy blood and tissue kit (Qiagen). Tissues of *D. haemobaphes* were subjected to DNA extraction via a phenol-chloroform method as described previously (6, 7). Heart and gill tissue samples from *C*. *maenas* were stored in ethanol and rinsed in molecular-grade water prior to homogenization using a FastPrep 24 homogenizer and a lysing matrix A tube (MP Biomedicals). DNA was extracted using Lifton’s buffer, followed by a phenol-chloroform method (35).

DNA libraries were generated using a Nextera XT DNA kit (Illumina) and sequenced using a v3 chemistry 600-cycle kit on a MiSeq sequencer (Illumina). For the sequencing data from *P. argu*s and *D. haemobaphes*, *de novo* assemblies of the paired-end reads were performed in SPAdes V3.5.0 (36) with default settings and *K* values equal to 21, 33, 55, 77, 99, and 127. Viral reads were assembled to form a single contig, and no additional steps were performed to remove the host reads. The sequence data from *C. maenas* were trimmed to remove low-quality bases and adapter sequences using Trim Galore! v0.4.0 (https://www.bioinformatics.babraham.ac.uk/projects/trim_galore/). Sequences were normalized and error corrected using BBNorm (BBTools v38.03) with the following parameters: ecc=t bits=16 prefilter. The reads were assembled *de novo* using Unicycler v0.4.4 (37) in “conservative” mode, and the error correction was switched off.

### Sequence analysis of the PaV1, CmV1, and DhV1 genomes.

Open reading frames (ORFs) in PaV1, CmV1, and DhV1 were predicted using GeneMark hmm version 3.25 with default parameters (38). To identify clusters of orthologous genes for the three viruses, the deduced amino acid sequences of the ORFs were searched against each other using BLASTP (39). Best hits from one genome to the others were collected to form pairs of orthologs and linked together into orthologous families. When a member of an orthologous cluster was missing in one of the viruses, a TBLASTN (39) search was performed against the respective genome translated in 6 frames using the cluster members from the other viruses as queries, in order to identify orthologs that might have been missed by GeneMark, possibly due to a frameshift. This search yielded 48 clusters of apparent orthologs (orthogroups), which included proteins from at least two of the three genomes. The ORF predictions were manually corrected using the coordinates of the proteins in the orthologous clusters. The resulting protein sequences were used as queries to search the NCBI nonredundant protein database using PSI-BLAST (39); the Conserved Domain Database (CDD) using RPS-BLAST (40); and the PDB, CDD, and Pfam databases using HHPred with default parameters (41). Sequences of every orthogroup were aligned using MUSCLE (42); the alignments were used as queries to search the PDB, CDD, and Pfam databases using HHPred; and orthologous clusters and individual proteins were annotated, wherever possible, based on these search results. Among the proteins not included in the orthologous clusters, only one received a functional annotation, namely, that encoded by gene 3 of PaV1 (T4 RNA ligase). Low-complexity segments in the NCLDV protein complements were identified using the SEGMASKER program of the NCBI BLAST^+^ suite, with a window size of 12, a trigger complexity threshold of 2.2 bits, and an extension complexity threshold of 2.5 bits. The total fractions of amino acids in the low-complexity segments were reported for each virus genome.

### Phylogenetic analyses.

Protein sequences were aligned using MUSCLE v3.7 with default parameters (39), and poorly aligned (low-information-content) positions were removed (43). Preliminary phylogenetic trees were constructed using the FastTree program with default parameters (44). The alignments of three conserved NCLDV proteins (DNAP, primase-helicase, and MCP) were concatenated and used for phylogenetic analysis with PhyML (45) (http://www.atgc-montpellier.fr/phyml-sms/). The best model identified by PhyML was LG+G+I+F (LG substitution model; gamma-distributed site rates with gamma shape parameter estimated from the alignment; the fraction of invariable sites estimated from the alignment; and empirical equilibrium frequencies). Support values were obtained using 100 bootstrap replicates.

### Data availability.

The analyzed data generated during this study can be downloaded from ftp://ftp.ncbi.nih.gov/pub/yutinn/Crustacean_viruses_2018. The genome sequences of PaV1, CmV1, and DhV1 have been deposited in the NCBI GenBank database under accession no. MN604017, MN604015, and MN604016, respectively.
