# Supplementary material for: A New Family of DNA Viruses Causing Disease in Crustaceans from Diverse Aquatic Biomes
Source: mBio. 2020 Jan 14;11(1):e02938-19. doi: 10.1128/mBio.02938-19 (PMC6960288; doi:10.1128/mBio.02938-19)
Supplement: FIG S1 [file mBio.02938-19-sf001.pdf]

packaging ATPase

cluster PaV1_21 aligned with representative Pitho-, Irido, Marseille-, and Poxviruses

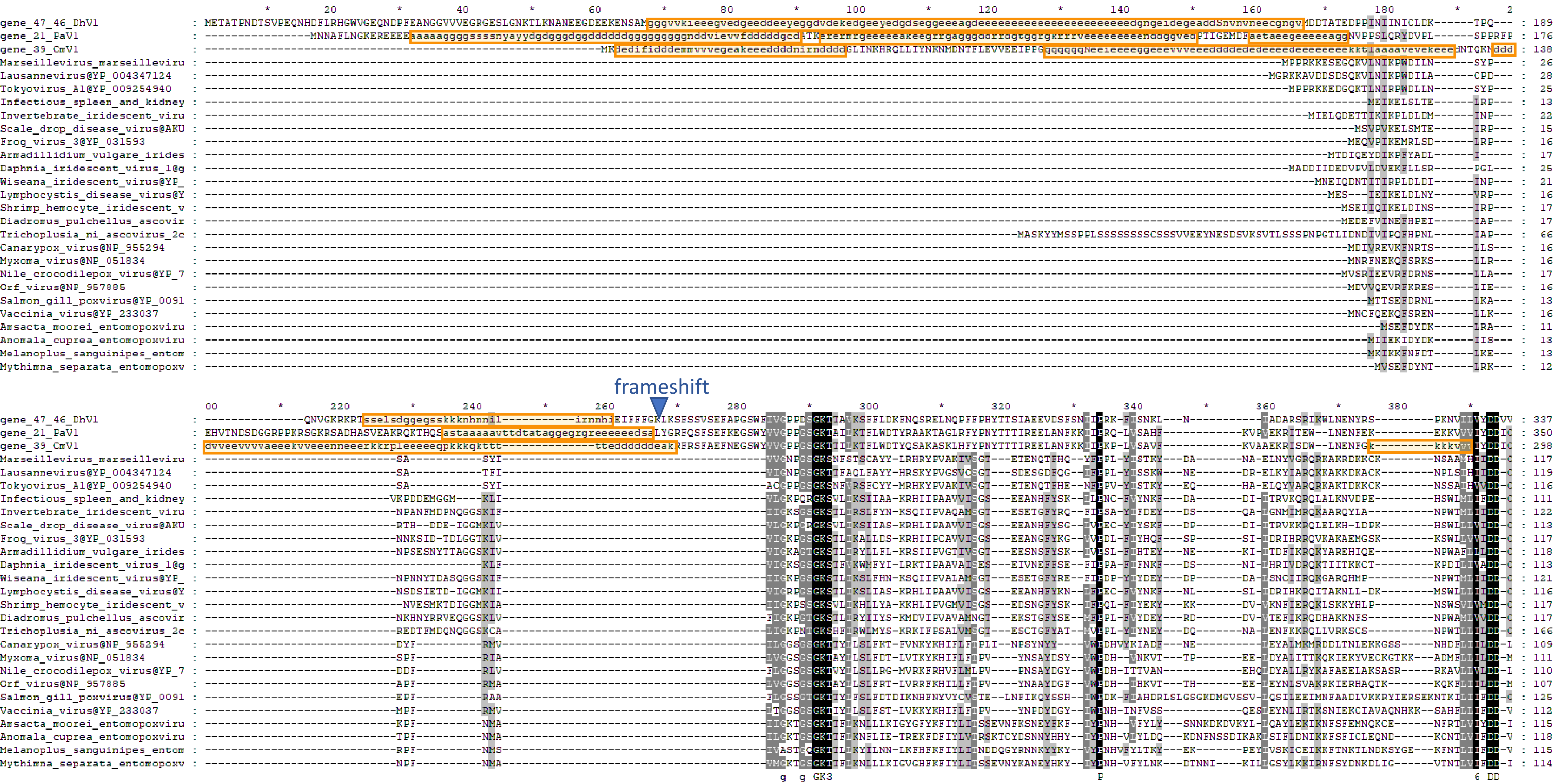
frameshift

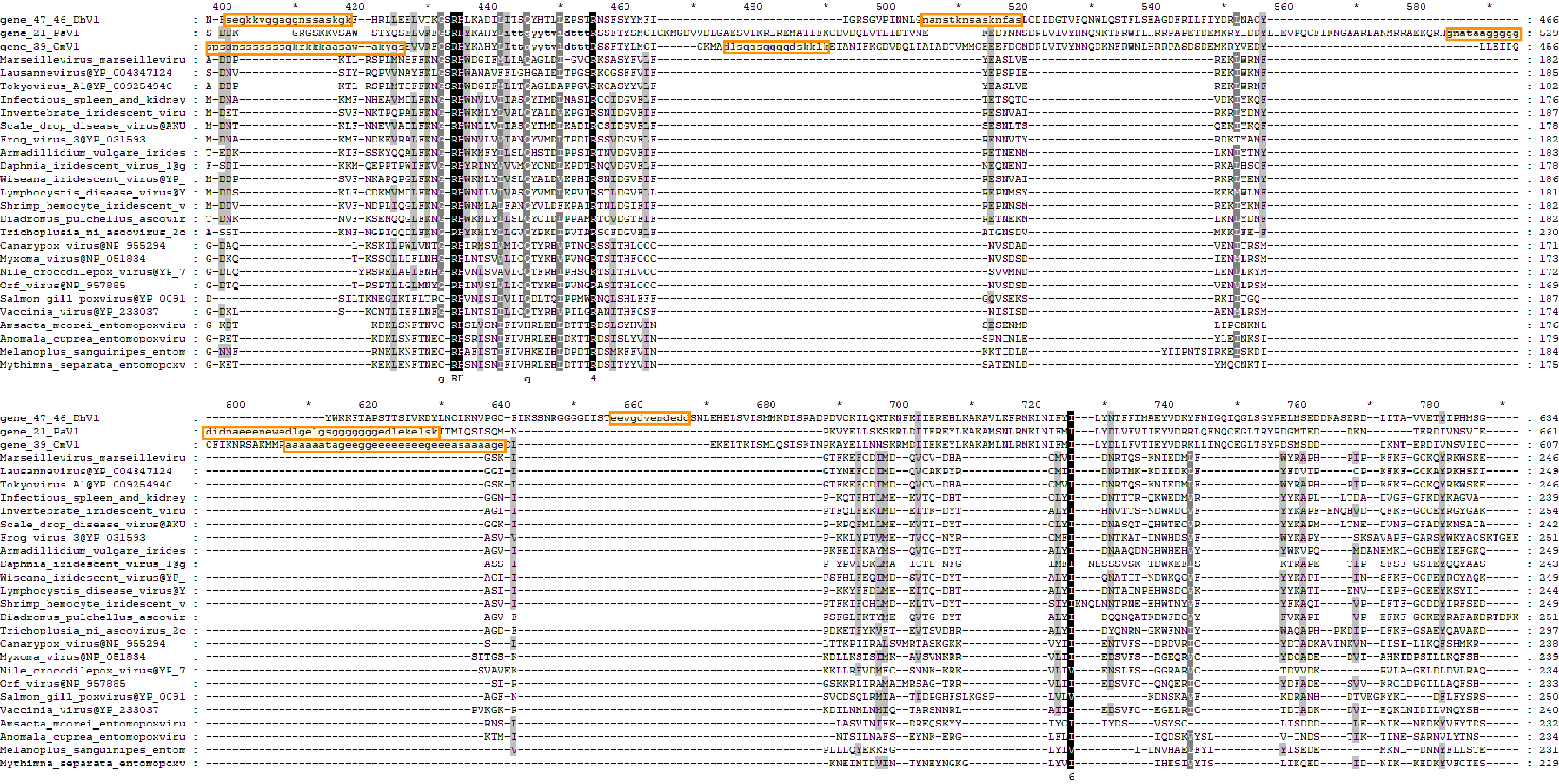

400 420 440 460 480 500 520 540 560 580
gene_47_46_DhV1 466
gene_21_PaV1 529
gene_39_CmV1 456
Marseillevirus_marseilleviru 182
Lausannevirus@YP_004347124 185
Tokyovirus_A1@YP_009254940 182
Infectious_spleen_and_kidney 176
Invertebrate_iridescent_viru 187
Scale_drop_disease_virus@AKU 178
Frog_virus_3@YP_031593 182
Armadillidium_vulgare_irides 183
Daphnia_iridescent_virus_1@g 178
Wiseana_iridescent_virus@YP_ 186
Lymphocystis_disease_virus@Y 181
Shrimp_hemocyte_iridescent_v 182
Diadromus_pulchellus_ascovir 182
Trichoplusia_ni_ascovirus_2c 230
Canarypox_virus@NP_955294 171
Myxoma_virus@NP_051834 173
Nile_crocodilepox_virus@YP_7 172
Orf_virus@NP_957885 169
Salmon_gill_poxvirus@YP_0091 187
Vaccinia_virus@YP_233037 174
Amsacta_moorei_entomopoxviru 176
Anomala_cuprea_entomopoxviru 179
Melanoplus_sanguinipes_entom 184
Mythimna_separata_entomopoxv 175
g RH q 4
600 620 640 660 680 700 720 740 760 780
gene_47_46_DhV1 634
gene_21_PaV1 661
gene_39_CmV1 607
Marseillevirus_marseilleviru 246
Lausannevirus@YP_004347124 249
Tokyovirus_A1@YP_009254940 246
Infectious_spleen_and_kidney 239
Invertebrate_iridescent_viru 254
Scale_drop_disease_virus@AKU 242
Frog_virus_3@YP_031593 251
Armadillidium_vulgare_irides 249
Daphnia_iridescent_virus_1@g 243
Wiseana_iridescent_virus@YP_ 249
Lymphocystis_disease_virus@Y 244
Shrimp_hemocyte_iridescent_v 249
Diadromus_pulchellus_ascovir 251
Trichoplusia_ni_ascovirus_2c 297
Canarypox_virus@NP_955294 238
Myxoma_virus@NP_051834 239
Nile_crocodilepox_virus@YP_7 234
Orf_virus@NP_957885 233
Salmon_gill_poxvirus@YP_0091 250
Vaccinia_virus@YP_233037 240
Amsacta_moorei_entomopoxviru 232
Anomala_cuprea_entomopoxviru 234
Melanoplus_sanguinipes_entom 231
Mythimna_separata_entomopoxv 229
6

Thymidylate or Deoxyguanosine kinase

cluster PaV1_15 aligned with nearest Pitho-, Irido, Marseilleviruses

taken from: ftp://ftp.ncbi.nih.gov/pub/yutinn/Loki_Castle_NCLDV_2018/PIM_clusters/PIM_afa/pim0066.afa

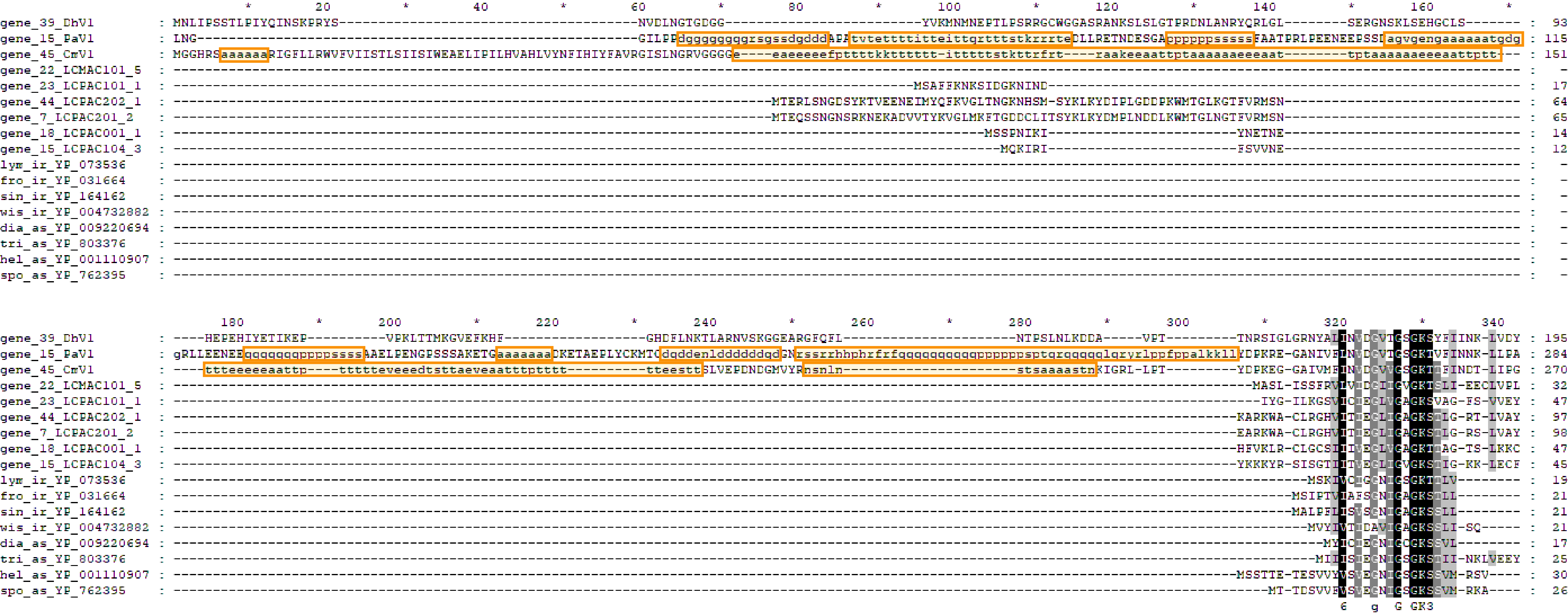
gene_39_DhV1
gene_15_PaV1
gene_45_CmV1
gene_22_LCMAC101_5
gene_23_LCPAC101_1
gene_44_LCPAC202_1
gene_7_LCPAC201_2
gene_18_LCPAC001_1
gene_15_LCPAC104_3
lym_ir_YP_073536
fro_ir_YP_031664
sin_ir_YP_164162
wis_ir_YP_004732882
dia_as_YP_009220694
tri_as_YP_803376
hel_as_YP_001110907
spo_as_YP_762395

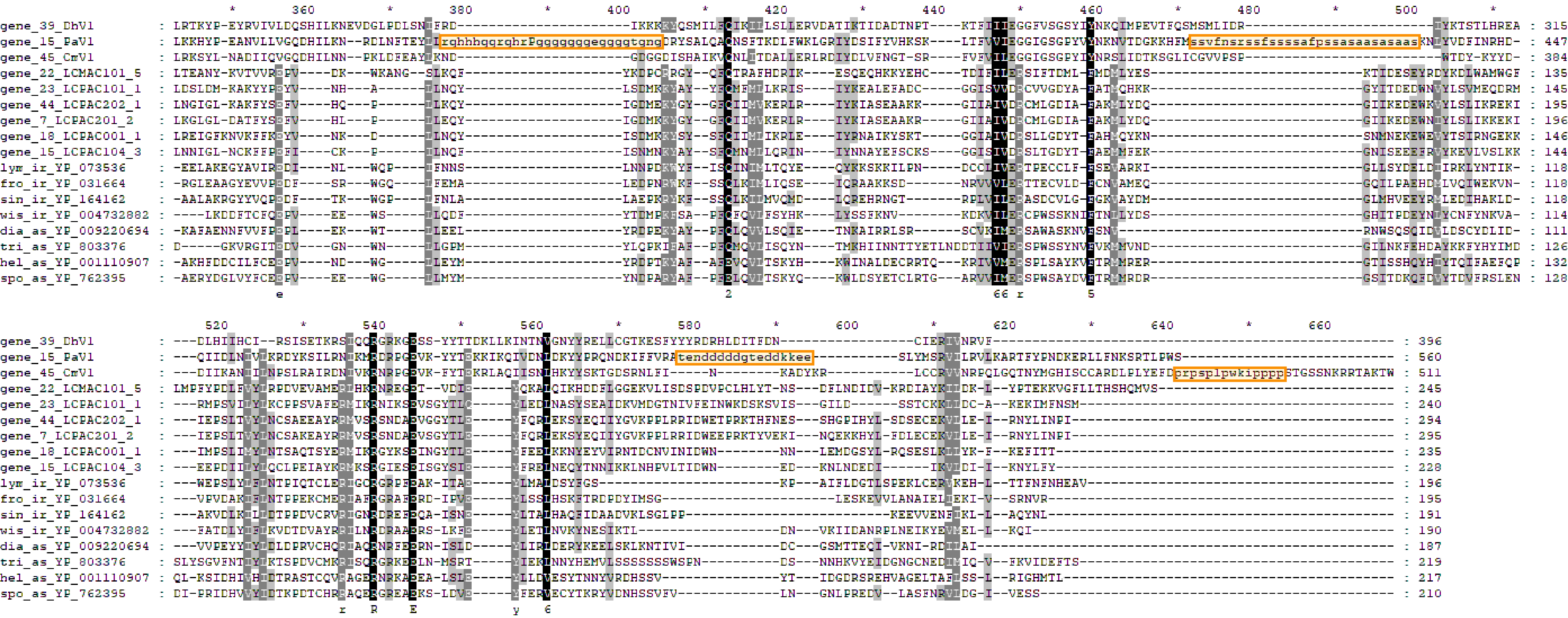
gene_39_DhV1
gene_15_PaV1
gene_45_CmV1
gene_22_LCMAC101_5
gene_23_LCPAC101_1
gene_44_LCPAC202_1
gene_7_LCPAC201_2
gene_18_LCPAC001_1
gene_15_LCPAC104_3
lym_ir_YP_073536
fro_ir_YP_031664
sin_ir_YP_164162
wis_ir_YP_004732882
dia_as_YP_009220694
tri_as_YP_803376
hel_as_YP_001110907
spo_as_YP_762395

RNApB; DNA-dependent RNA polymerase subunit RPB2

cluster PaV1_25 aligned with representative Pitho-, Irido, Marseilleviruses

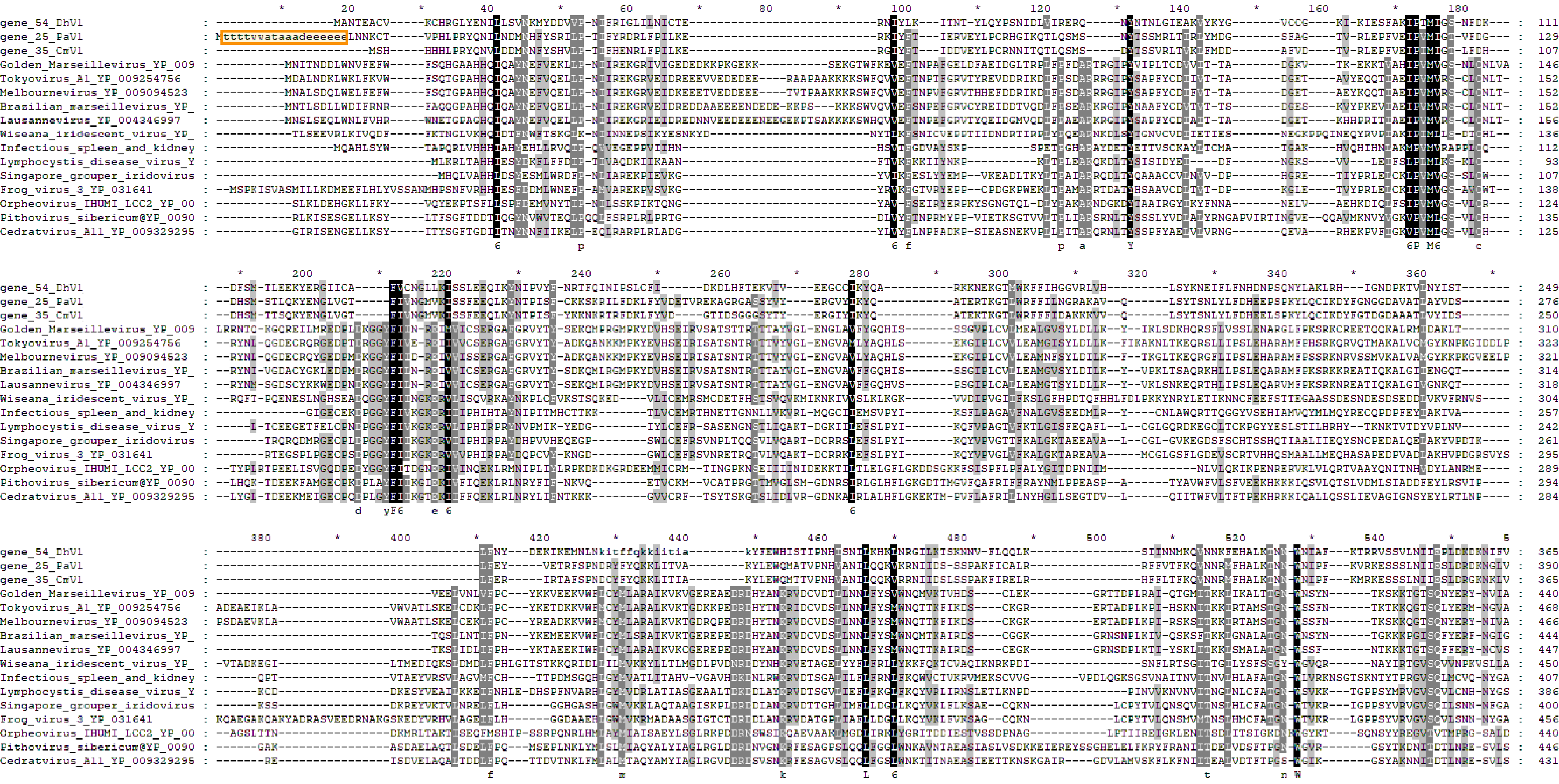
gene_54_DhV1
gene_25_PaV1
gene_35_CmV1
Golden_Marseillevirus_YP_009
Tokyovirus_A1_YP_009254756
Melbournevirus_YP_009094523
Brazilian_marseillevirus_YP_
Lausannevirus_YP_004346997
Wiseana_iridescent_virus_YP_
Infectious_spleen_and_kidney
Lymphocystis_disease_virus_Y
Singapore_grouper_iridovirus
Frog_virus_3_YP_031641
Orpheovirus_IHUMI_LCC2_YP_00
Pithovirus_sibericum@YP_0090
Cedratvirus_A11_YP_009329295

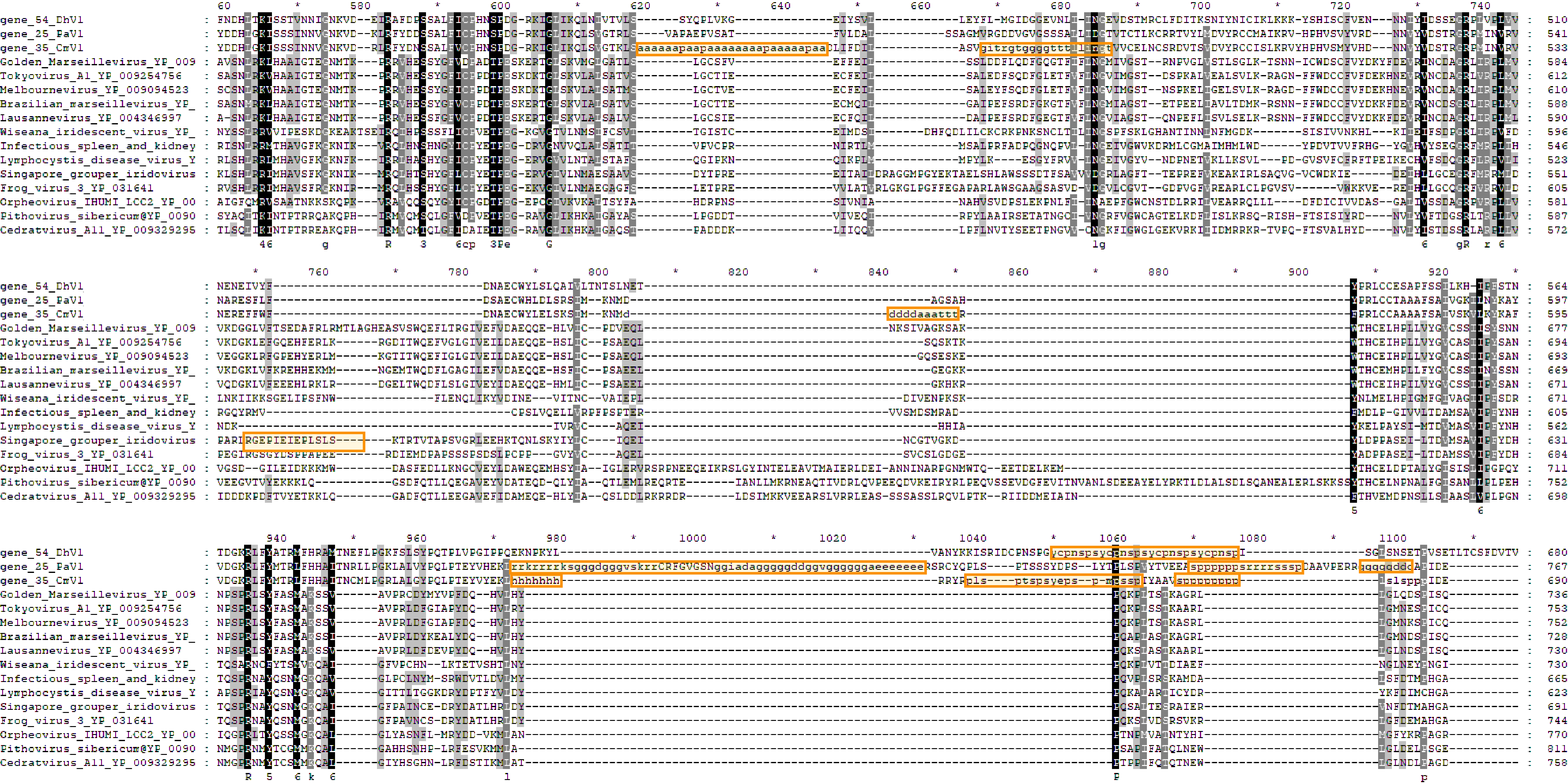

gene_54_DhV1
gene_25_PaV1
gene_35_CmV1
Golden_Marseillevirus_YP_009
Tokyovirus_A1_YP_009254756
Melbournevirus_YP_009094523
Brazilian_marseillevirus_YP_
Lausannevirus_YP_004346997
Wiseana_iridescent_virus_YP_
Infectious_spleen_and_kidney
Lymphocystis_disease_virus_Y
Singapore_grouper_iridovirus
Frog_virus_3_YP_031641
Orpheovirus_IHUMI_LCC2_YP_00
Pithovirus_sibericum@YP_0090
Cedratvirus_A11_YP_009329295

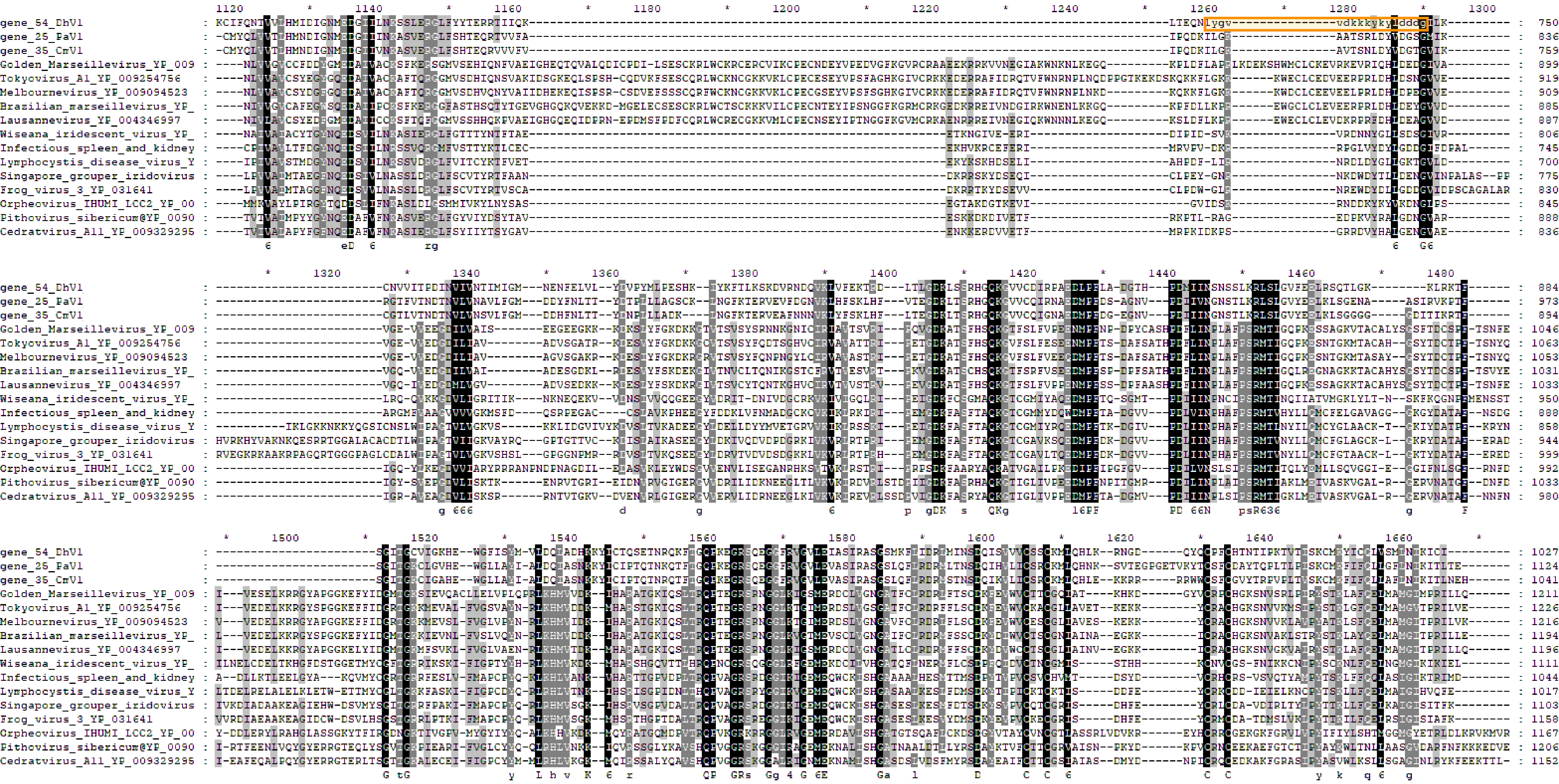

1120 1140 1160 1180 1200 1220 1240 1260 1280 1300
1320 1340 1360 1380 1400 1420 1440 1460 1480
1500 1520 1540 1560 1580 1600 1620 1640 1660
gene_54_DhV1
gene_25_PaV1
gene_35_CmV1
Golden_Marseillevirus_YP_009
Tokyovirus_A1_YP_009254756
Melbournevirus_YP_009094523
Brazilian_marseillevirus_YP_
Lausannevirus_YP_004346997
Wiseana_iridescent_virus_YP_
Infectious_spleen_and_kidney
Lymphocystis_disease_virus_Y
Singapore_grouper_iridovirus
Frog_virus_3_YP_031641
Orpheovirus_IHUMI_LCC2_YP_00
Pithovirus_sibericum@YP_0090
Cedratvirus_A11_YP_009329295
750 836 759 899 919 909 885 887 806 745 700 775 830 845 888 836
884 973 894 1046 1063 1053 1031 1033 950 888 858 944 999 992 1033 980
1027 1124 1041 1211 1226 1216 1194 1196 1111 1044 1017 1103 1158 1167 1206 1152
